# Crystal structure of poly[[di­aqua­tetra-μ_2_-cyanido-platinum(II)iron(II)] methanol 4/3-solvate]: a three-dimensional Hofmann clathrate analogue

**DOI:** 10.1107/S2056989022000573

**Published:** 2022-01-25

**Authors:** Volodymyr M. Hiiuk, Vasyl Mykhailovych, Sergiu Shova, Irina A. Golenya, Il’ya A. Gural’skiy

**Affiliations:** a Department of Chemistry, Taras Shevchenko National University of Kyiv, 64 Volodymyrska St, 01601 Kyiv, Ukraine; b UkrOrgSyntez Ltd, 67 Chervonotkatska St, 02094 Kyiv, Ukraine; c Faculty of Natural Sciences, National University of Kyiv-Mohyla Academy, 2 Skovorody St, 04070 Kyiv, Ukraine; d Faculty of Electrical Engineering and Computer Science & Research Center, MANSiD, Stefan cel Mare University, 13 Universitatii St., 720229 Suceava, Romania; e Department of Inorganic Polymers, "Petru Poni", Institute of Macromolecular Chemistry, Romanian Academy of Science, Aleea Grigore Ghica Voda 41-A, Iasi 700487, Romania

**Keywords:** coordination compounds, iron(II), clathrate, platinum, cyanido ligand, crystal structure

## Abstract

In the title polymeric coordination compound, {[FePt(CN)_4_(H_2_O)_2_]·1.33CH_3_OH}_*n*_, the Fe^II^ cation (site symmetry 4/*mm.m*) is coordinated by the N atoms of four cyanide anions (CN^−^) and the O atoms of two water mol­ecules, forming a nearly regular [FeN_4_O_2_] octa­hedron. According the Fe—N and Fe—O bond lengths, the Fe^II^ atom is in the high-spin state. The cyanide anions act in a bridging manner to connect the Fe^II^ and Pt^II^ atoms. The [Pt(CN)_4_]^2–^ moieties (Pt with site symmetry 4/*mm.m*) have a perfect square-planar shape. The latter anion is located perpendicular to the FeN_4_ plane, thus ensuring the creation of a three-dimensional framework. The crystal structure features methanol solvent mol­ecules of which 4/3 were located per Fe^II^ cation. These solvent mol­ecules are located in hexa­gonal pores; they inter­act with coordinating water mol­ecules through weak hydrogen bonds. Other guest mol­ecules could not be modelled in a satisfactory way and their contribution to the scattering was removed by a mask procedure.

## Abstract 2

The crystal structure of the title polymeric coordination compound, {[FePt(CN)_4_(H_2_O)_2_]·1.33CH_3_OH}_*n*_, features a framework structure with pores in which disordered methanol solvent mol­ecules are situated.

## Chemical context

Cyanide-based complexes form a large group of coordination compounds, which can offer numerous structures and functionalities. As a result of the ability of the cyanide anion to act in a bridging way, this group often links two different metal cations, enabling the formation of one-, two- or three-dimensional frameworks. The beginning of the investigation of cyanide-based complexes dates back to the 18th century when Prussian blue was discovered (Dacarro *et al.*, 2018 ▸). Since then, hundreds of cyanide-based complexes have been obtained and proven to be efficient as mol­ecular magnets, in separation, condensation, storage, catalysis, polymer synthesis, switching, *etc* (Zakaria & Chikyow, 2017 ▸).

Among all cyanide-based complexes, Hofmann clathrate analogues attract considerable attention. This is a group of polymeric coordination complexes with general formula [*M*(*L*)_*x*_{*M*′(CN)_*y*_}_*z*_·*n*(guest/solvent) where *M* has an octa­hedral coordination environment with two *L* ligands in axial positions and four N atoms of bridging cyanide groups in equatorial positions, which link *M* and *M*′ metals into infinite layers (Powell & Rayner, 1949 ▸; Hofmann & Küspert, 1897 ▸). If the *L* ligand is bridging as well (*e.g.* pyrazine), the creation of a three-dimensional framework is observed (Niel *et al.*, 2001 ▸). In addition, the chemical composition of Hofmann clathrates can easily be modified by variation of the guest/solvent mol­ecules.

One of the attractive properties of Hofmann clathrate analogues is the ability of some complexes of this class to undergo spin crossover under the influence of external stimuli (Carmen Muñoz & Real, 2011 ▸; Kucheriv *et al.*, 2021 ▸). The change of spin state can be observed in complexes of general formula [Fe(*L*)_*x*_{*M*′(CN)_*y*_}_*z*_] where *L* = azine or azole ligand, *M*′ = Cu, Ag, Au for *y* = 2, *z* = 2, and *M*′ = Ni, Pt, Pd for *y* = 4, *z* = 1 (Shylin *et al.*, 2020 ▸; Kuzevanova *et al.*, 2021 ▸). 
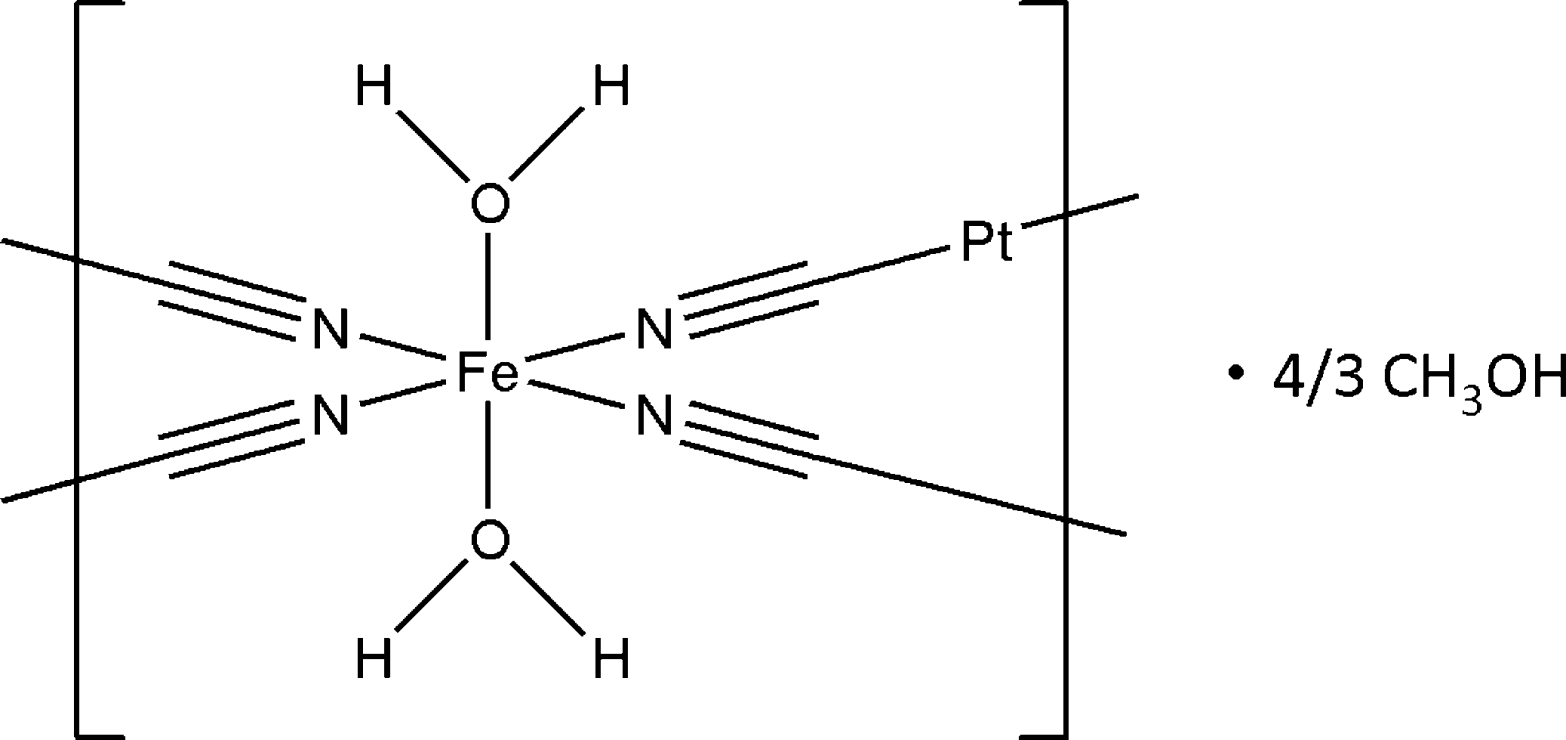


In this paper we report a {[FePt(CN)_4_(H_2_O)_2_]·4/3CH_3_OH}_*n*_ coordination polymer with a non-classical Hofmann-type framework.

## Structural commentary

**Figure 1:**
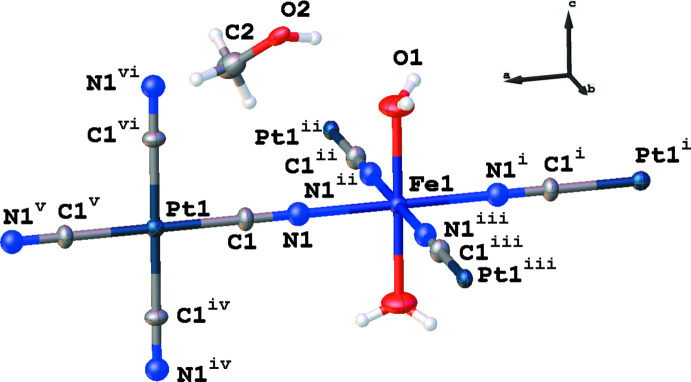
A fragment of the crystal structure of the title compound showing the atom-labelling scheme. Displacement ellipsoids are drawn at the 50% probability level. [Symmetry codes: (i) 1 − *x*, 1 − *y*, +*z*; (ii) *y*, 1 − *x*, +*z*; (iii) 1 − *y*, *x*, *z*; (iv) 1 − *z*, *y*, −1 + *x*; (v) 2 − *x*, 1 − *y*, *z*; (vi) 1 + *z*, *y*, 1 − *x*; (vii) *x*, 1 − *y*, −*z*].

**Figure 2:**
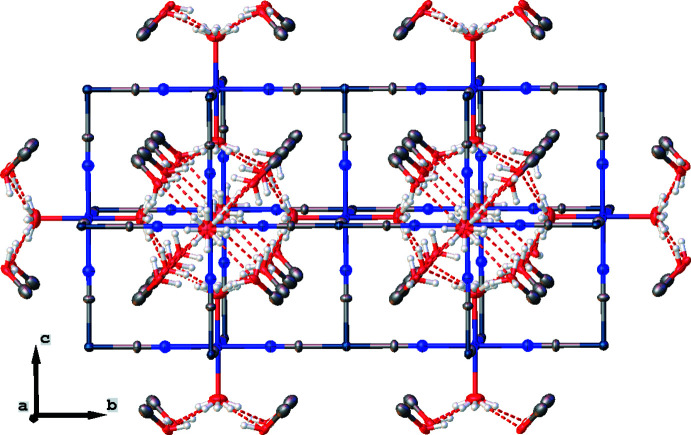
View of the crystal structure of the title compound along the *a* axis showing the three-dimensional coordination framework. Hydrogen bonds are shown as red dashed lines. Hydrogen atoms of the methyl group of the methanol solvent mol­ecules are omitted for clarity.

The Fe^II^ cation (site symmetry 4/*mm.m*; Wyckoff position 3*c*) exists in an [FeN_4_O_2_] coordination environment (Fig. 1 ▸), which is formed by the N atoms of four cyanide anions in equatorial positions [Fe1—N1 = 2.155 (18) Å] and the O atoms of two water mol­ecules in axial positions [Fe1— O1 = 2.15 (2) Å]. The similar lengths of the Fe—O and Fe—N bonds provide an almost ideal octa­hedral environment. The Fe^II^—O and Fe^II^—N bond lengths indicate that, at the temperature of the diffraction study, Fe^II^ is in the high-spin state. The cyanide anions connect the Fe^II^ and Pt^II^ atoms, whereby the latter (site symmetry 4/*mm.m*; Wyckoff position 3*d*) has a perfect square-planar environment with a Pt1—C1 bond length of 1.953 (17) Å. Contrary to classical Hofmann clathrate arrangements (Kucheriv *et al.*, 2021 ▸), the tetra­cyanidoplatinate(II) anions in the title compound are located perpendicular to the FeN_4_ plane, which ensures the creation of a three-dimensional framework (Fig. 2 ▸). As a result of the cubic symmetry of the crystal structure, no deviation from linearity is observed for the Fe–N–C–Pt fragments.

**Figure 3:**
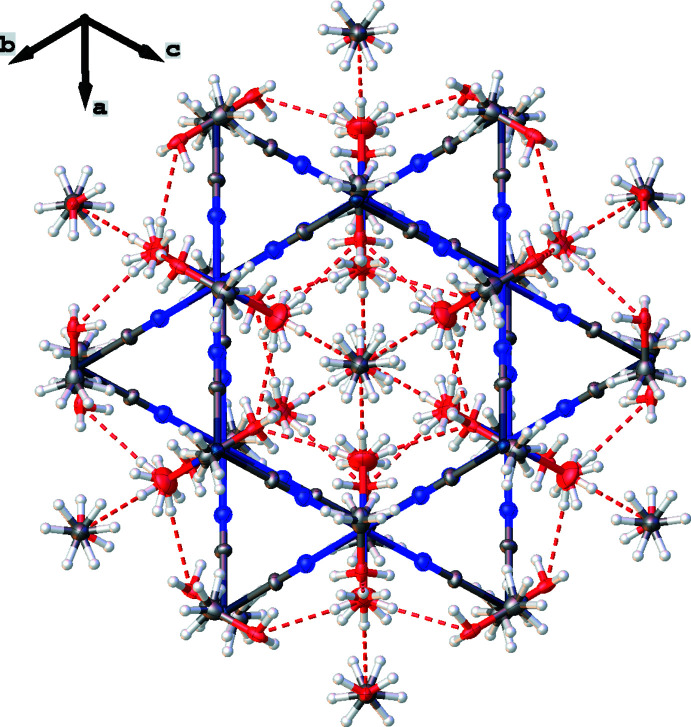
View of the crystal structure of the title compound showing the methanol solvent mol­ecules, which are located in hexa­gonal pores. Hydrogen bonds are shown as red dashed lines.

The title compound incorporates 4/3 methanol solvent mol­ecules per [FePt(CN)_4_(H_2_O)_2_] unit, which are located in hexa­gonal pores (Fig. 3 ▸) and inter­act with the coordinating water mol­ecules through O—H⋯O hydrogen bonds (Table 1 ▸). The framework features some additional highly disordered guest mol­ecules, which could not be modelled satisfactorily. Their contribution to the scattering was removed with a mask procedure implemented in *OLEX2* (Dolomanov *et al.*, 2009 ▸). These disordered guest mol­ecules reside in two types of void with total volumes of 138.3 and 20.3 Å^3^ corresponding to 36.4 and 2.6 electrons, respectively.

In comparison, two similar coordination compounds, *viz*. [Fe(H_2_O)_2_{Pt(CN)_4_]·2acetone (Kuzevanova *et al.*, 2019 ▸) and [Fe(H_2_O)_2_{Ni(CN)_4_}]·2dioxane (Yuge *et al.*, 1997 ▸), form infin­ite {Fe*M* ^II^(CN)_4_}_∞_ layers. The size of the available voids between the cyanidometallate layers in these two compounds allows the acetone or dioxane mol­ecules to rotate freely, thus leading to a high disorder of the solvent. Both of these compounds, as well as the title compound, represent spectacular examples of how variation of the guest/solvent mol­ecule can significantly influence the crystal structure of the coord­ination framework. Whereas small mol­ecules of methanol can fit inside the hexa­gonal pores of a three-dimensional framework, bulkier acetone or dioxane mol­ecules cannot be placed there, thus inducing the creation of layers.

## Database survey

A survey of the Cambridge Structural Database (Version 5.40; Groom *et al.*, 2016 ▸) revealed 106 framework structures containing Fe–N–C–Pt fragments. Among them there are three structures with an [FeN_5_O] coordination environment [AMIJEN (Kucheriv *et al.*, 2016 ▸), ZOHBEG and ZOHBIK (Wong *et al.*, 2019 ▸)], three structures with an [FeN_4_O_2_] coord­ination environment [CEMJAI (Piñeiro-López *et al.*, 2017 ▸), HOCRAU (Zhang *et al.*, 2014 ▸) and OKITAF (Haraguchi *et al.*, 2016 ▸)] and three structures that have two different Fe^II^ cations forming [FeN_4_O_2_] and [FeN_6_] octa­hedra [AMIJOX (Kucheriv *et al.*, 2016 ▸) and VOKLIS, VOKLIS01 (Sciortino *et al.*, 2014 ▸)].

## Synthesis and crystallization

Crystals of the title compound were grown by slow diffusion between three layers in a 3 ml tube. The first layer was a solution of K_2_[Pt(CN)_4_] (0.02 mmol) in water (0.5 ml), the second was a mixture of water/methanol (1:1, 1.5 ml) and the third layer was a solution of Fe(OTs)_2_·6H_2_O (0.02 mmol) (OTs = *p*-toluene­sulfonate) in methanol (0.5 ml). After two weeks, colourless crystals grew in the middle layer; these were collected and maintained under the mother solution until measured.

## Refinement

Crystal data, data collection and structure refinement details are summarized in Table 2 ▸. H atoms of water mol­ecules and the methanol OH group were placed at calculated positions and refined as riding on the bonded O atom. The occupancy of methanol atoms was refined and found to be equal to approximately 0.5 and later restrained to half-occupancy. As a result of symmetry restrictions, H atoms of the water mol­ecule are disordered over four positions and were constrained to have an occupancy of 1/4. The three H atoms of the methyl group are disordered over two sets of sites, and were refined as for an idealized methyl group and were allowed to rotate about the O—C bond. The H atom of the OH group is disordered over three sites. Its occupancy was restrained to coincide with half-occupancy of the complete mol­ecule.

## Supplementary Material

Crystal structure: contains datablock(s) I. DOI: 10.1107/S2056989022000573/wm5624sup1.cif

Structure factors: contains datablock(s) I. DOI: 10.1107/S2056989022000573/wm5624Isup2.hkl

CCDC reference: 2142647

Additional supporting information:  crystallographic information; 3D view; checkCIF report

## Figures and Tables

**Table 1:** Hydrogen-bond geometry (Å, °)

*D*—H⋯*A*	*D*—H	H⋯*A*	*D*⋯*A*	*D*—H⋯*A*
O1—H1*A*⋯O2	0.82	2.20	3.020 (18)	175

**Table 2:** Experimental details

Crystal data	
Chemical formula	[FePt(CN)_4_(H_2_O)_2_]·1.33CH_4_O
*M*_r_	433.77
Crystal system, space group	Cubic, *P* *m*\overline{3}*m*
Temperature (K)	293
*a* (Å)	10.5089 (3)
*V* (Å^3^)	1160.56 (10)
*Z*	3
Radiation type	Mo *K*α
μ (mm^−1^)	9.96
Crystal size (mm)	0.04 × 0.04 × 0.04

Data collection	
Diffractometer	Xcalibur, Eos
Absorption correction	Multi-scan (*CrysAlis PRO*; Rigaku OD, 2021 ▸)
*T*_min_, *T*_max_	0.930, 1.000
No. of measured, independent and observed [*I* > 2σ(*I*)] reflections	2210, 336, 273
*R*_int_	0.091
(sin θ/λ)_max_ (Å^−1^)	0.688

Refinement	
*R*[*F* ^2^ > 2σ(*F* ^2^)], *wR*(*F* ^2^), *S*	0.055, 0.133, 1.07
No. of reflections	336
No. of parameters	23
No. of restraints	13
H-atom treatment	H-atom parameters constrained
Δρ_max_, Δρ_min_ (e Å^−3^)	2.15, −1.36

Computer programs: *CrysAlis PRO* (Rigaku OD, 2021 ▸), *SHELXT* (Sheldrick, 2015*a* ▸), *SHELXL* (Sheldrick, 2015*b* ▸) and *OLEX2* (Dolomanov *et al.*, 2009 ▸).

## supplementary crystallographic information

### Crystal data

**Table:**

[FePt(CN)_4_(H_2_O)_2_]·1.33CH_4_O	Mo *K*α radiation, λ = 0.71073 Å
*M**_r_* = 433.77	Cell parameters from 449 reflections
Cubic, *P**m*3*m*	θ = 1.9–21.7°
*a* = 10.5089 (3) Å	µ = 9.96 mm^−^^1^
*V* = 1160.56 (10) Å^3^	*T* = 293 K
*Z* = 3	Cube, clear intense colourless
*F*(000) = 600	0.04 × 0.04 × 0.04 mm
*D*_x_ = 1.862 Mg m^−^^3^	

### Data collection

**Table:**

Xcalibur, Eos diffractometer	336 independent reflections
Radiation source: fine-focus sealed X-ray tube, Enhance (Mo) X-ray Source	273 reflections with *I* > 2σ(*I*)
Graphite monochromator	*R*_int_ = 0.091
Detector resolution: 16.1593 pixels mm^-1^	θ_max_ = 29.3°, θ_min_ = 1.9°
ω scans	*h* = −9→8
Absorption correction: multi-scan (CrysAlisPro; Rigaku OD, 2021)	*k* = −14→14
*T*_min_ = 0.930, *T*_max_ = 1.000	*l* = −5→14
2210 measured reflections	

### Refinement

**Table:**

Refinement on *F*^2^	13 restraints
Least-squares matrix: full	Hydrogen site location: mixed
*R*[*F*^2^ > 2σ(*F*^2^)] = 0.055	H-atom parameters constrained
*wR*(*F*^2^) = 0.133	*w* = 1/[σ^2^(*F*_o_^2^) + (0.0611*P*)^2^] where *P* = (*F*_o_^2^ + 2*F*_c_^2^)/3
*S* = 1.07	(Δ/σ)_max_ < 0.001
336 reflections	Δρ_max_ = 2.15 e Å^−^^3^
23 parameters	Δρ_min_ = −1.36 e Å^−^^3^

### Special details

**Table:**

Geometry. All esds (except the esd in the dihedral angle between two l.s. planes) are estimated using the full covariance matrix. The cell esds are taken into account individually in the estimation of esds in distances, angles and torsion angles; correlations between esds in cell parameters are only used when they are defined by crystal symmetry. An approximate (isotropic) treatment of cell esds is used for estimating esds involving l.s. planes.

### Fractional atomic coordinates and isotropic or equivalent isotropic displacement parameters (Å^2^)

**Table:**

	*x*	*y*	*z*	*U*_iso_*/*U*_eq_	Occ. (<1)
Pt1	1.000000	0.500000	0.000000	0.0374 (5)	
Fe1	0.500000	0.500000	0.000000	0.0395 (12)	
N1	0.7051 (17)	0.500000	0.000000	0.059 (4)	
C1	0.8141 (17)	0.500000	0.000000	0.047 (4)	
O1	0.500000	0.500000	0.2049 (19)	0.112 (9)	
H1A	0.549690	0.450310	0.238738	0.168*	0.25
H1B	0.423680	0.500000	0.233848	0.168*	0.25
O2	0.6888 (17)	0.3112 (17)	0.3112 (17)	0.049 (7)	0.5
H2	0.610990	0.294000	0.294000	0.073*	0.1667
C2	0.7666 (19)	0.2334 (19)	0.2334 (19)	0.088 (14)	0.5
H2A	0.728153	0.224628	0.151003	0.132*	0.0833
H2B	0.775372	0.151003	0.271847	0.132*	0.0833
H2C	0.848997	0.271847	0.224628	0.132*	0.0833

### Atomic displacement parameters (Å^2^)

**Table:**

	*U*^11^	*U*^22^	*U*^33^	*U*^12^	*U*^13^	*U*^23^
Pt1	0.0420 (6)	0.0282 (7)	0.0420 (6)	0.000	0.000	0.000
Fe1	0.0399 (16)	0.0399 (16)	0.039 (2)	0.000	0.000	0.000
N1	0.060 (11)	0.054 (10)	0.064 (11)	0.000	0.000	0.000
C1	0.032 (9)	0.046 (10)	0.063 (12)	0.000	0.000	0.000
O1	0.144 (15)	0.144 (15)	0.047 (14)	0.000	0.000	0.000
O2	0.049 (7)	0.049 (7)	0.049 (7)	−0.019 (7)	−0.019 (7)	0.019 (7)
C2	0.088 (14)	0.088 (14)	0.088 (14)	−0.021 (11)	−0.021 (11)	0.021 (11)

### Geometric parameters (Å, º)

**Table:**

Pt1—C1^i^	1.953 (17)	O1—H1A	0.8198
Pt1—C1^ii^	1.953 (17)	O1—H1A^ix^	0.8198
Pt1—C1^iii^	1.953 (17)	O1—H1B^ix^	0.8579
Pt1—C1	1.953 (17)	O1—H1B^x^	0.8579
Fe1—N1	2.155 (18)	O1—H1B^viii^	0.8579
Fe1—N1^iv^	2.155 (18)	O1—H1B	0.8579
Fe1—N1^v^	2.155 (18)	O2—H2	0.8565
Fe1—N1^vi^	2.155 (18)	O2—H2^xi^	0.8565
Fe1—O1^v^	2.15 (2)	O2—H2^xii^	0.8565
Fe1—O1	2.15 (2)	O2—C2	1.42 (2)
N1—C1	1.15 (2)	C2—H2A	0.9600
O1—H1A^vii^	0.8198	C2—H2B	0.9600
O1—H1A^viii^	0.8198	C2—H2C	0.9600
			
C1^i^—Pt1—C1^iii^	180.0	H1A—O1—H1A^ix^	79.1
C1^iii^—Pt1—C1	90.0	H1A^viii^—O1—H1B^x^	116.2
C1^i^—Pt1—C1	90.0	H1A—O1—H1B^x^	41.4
C1^i^—Pt1—C1^ii^	90.0	H1A^vii^—O1—H1B^viii^	116.2
C1^iii^—Pt1—C1^ii^	90.0	H1A^vii^—O1—H1B^ix^	41.4
C1^ii^—Pt1—C1	180.0	H1A—O1—H1B	116.2
N1—Fe1—N1^v^	180.0	H1A^vii^—O1—H1B^x^	41.4
N1^vi^—Fe1—N1^iv^	180.0	H1A—O1—H1B^ix^	116.2
N1^v^—Fe1—N1^iv^	90.0	H1A—O1—H1B^viii^	41.4
N1—Fe1—N1^vi^	90.0	H1A^ix^—O1—H1B^x^	116.2
N1^v^—Fe1—N1^vi^	90.0	H1A^ix^—O1—H1B^viii^	41.4
N1—Fe1—N1^iv^	90.0	H1A^viii^—O1—H1B^viii^	116.2
O1^v^—Fe1—N1^vi^	90.0	H1A^viii^—O1—H1B^ix^	41.4
O1^v^—Fe1—N1^iv^	90.0	H1A^ix^—O1—H1B^ix^	116.2
O1—Fe1—N1^v^	90.0	H1B—O1—H1A^vii^	116.2
O1—Fe1—N1^vi^	90.0	H1B—O1—H1A^ix^	41.4
O1—Fe1—N1^iv^	90.0	H1B—O1—H1A^viii^	41.4
O1^v^—Fe1—N1	90.0	H1B^ix^—O1—H1B^viii^	138.4
O1—Fe1—N1	90.0	H1B^viii^—O1—H1B^x^	82.8
O1^v^—Fe1—N1^v^	90.0	H1B—O1—H1B^viii^	82.8
O1^v^—Fe1—O1	180.0	H1B—O1—H1B^ix^	82.8
C1—N1—Fe1	180.0	H1B—O1—H1B^x^	138.4
N1—C1—Pt1	180.0	H1B^ix^—O1—H1B^x^	82.8
Fe1—O1—H1A^viii^	115.740 (1)	H2^xi^—O2—H2^xii^	111.0
Fe1—O1—H1A^ix^	115.740 (1)	H2—O2—H2^xii^	111.0
Fe1—O1—H1A	115.7	H2—O2—H2^xi^	111.0
Fe1—O1—H1A^vii^	115.740 (2)	C2—O2—H2	107.9
Fe1—O1—H1B	110.8	C2—O2—H2^xii^	107.857 (3)
Fe1—O1—H1B^ix^	110.8	C2—O2—H2^xi^	107.857 (8)
Fe1—O1—H1B^x^	110.799 (2)	O2—C2—H2A	109.5
Fe1—O1—H1B^viii^	110.8	O2—C2—H2B	109.5
H1A^ix^—O1—H1A^viii^	79.1	O2—C2—H2C	109.5
H1A—O1—H1A^viii^	128.5	H2A—C2—H2B	109.5
H1A—O1—H1A^vii^	79.1	H2A—C2—H2C	109.5
H1A^ix^—O1—H1A^vii^	128.5	H2B—C2—H2C	109.5
H1A^viii^—O1—H1A^vii^	79.1		

Symmetry codes: (i) −*z*+1, −*y*+1, *x*−1; (ii) −*x*+2, −*y*+1, −*z*; (iii) *z*+1, *y*, −*x*+1; (iv) −*y*+1, −*x*+1, *z*; (v) −*x*+1, −*y*+1, −*z*; (vi) *y*, *x*, −*z*; (vii) −*y*+1, *x*, *z*; (viii) *y*, *x*, *z*; (ix) *y*, −*x*+1, *z*; (x) −*x*+1, −*y*+1, *z*; (xi) −*z*+1, −*x*+1, *y*; (xii) −*y*+1, *z*, −*x*+1.

### Hydrogen-bond geometry (Å, º)

**Table:**

*D*—H···*A*	*D*—H	H···*A*	*D*···*A*	*D*—H···*A*
O1—H1*A*···O2	0.82	2.20	3.020 (18)	175
